# Deep Neck Infection: Atypical Presentation of Papillary Thyroid Cancer

**DOI:** 10.1155/2021/1479201

**Published:** 2021-10-18

**Authors:** Apichana Mahattanapreut, Rangsima Aroonroch, Chalermchai Chintrakarn, Chutintorn Sriphrapradang

**Affiliations:** ^**1**^ Division of Endocrinology and Metabolism, Department of Medicine, Faculty of Medicine Ramathibodi Hospital, Mahidol University, Bangkok 10400, Thailand; ^2^Department of Pathology, Faculty of Medicine Ramathibodi Hospital, Mahidol University, Bangkok 10400, Thailand; ^3^Department of Otolaryngology, Faculty of Medicine Ramathibodi Hospital, Mahidol University, Bangkok 10400, Thailand

## Abstract

Deep neck infection is defined as an infectious process in the potential spaces and fascial plane of the neck which may result in a fatal complication. Prompt drainage and broad-spectrum antibiotics are the mainstays of treatment. Deep neck infection as the initial presentation of primary head and neck cancer is not common. Nevertheless, head and neck squamous cell carcinoma is the most common primary head and neck cancer, which could present with cervical metastasis and subsequently becomes infected. Papillary thyroid cancer has a naturally indolent course, and most patients present with a thyroid nodule. However, deep neck infection could be an uncommon presentation of papillary thyroid cancer which may obscure the diagnosis of underlying malignancy. This case report aims to present a rare presentation of papillary thyroid cancer which needs meticulous evaluation. Moreover, the pathological examination should be performed in all cases of deep neck infection for early detection and management of underlying papillary thyroid cancer.

## 1. Introduction

Deep neck infection (DNI) is a serious condition that can potentially be life-threatening. The infectious process mostly originates from other primary sites (e.g., odontogenic source and upper respiratory tract) and invades potential spaces and fascial planes of the neck. The bacteriology is usually mixed with aerobic and anaerobic bacteria [1]. Cystic nodal metastasis of primary head and neck cancer could become infected and present as a DNI. Although head and neck squamous cell carcinomas often present with cervical metastases, DNI as the initial presentation of primary head and neck cancer is not common [2]. Central necrosis and direct tumor extension can also develop DNI [3].

Papillary thyroid cancer (PTC) is well-differentiated thyroid cancer and the most common type of thyroid cancer. Generally, PTC is a disease with an indolent course with an excellent overall prognosis [4]. The typical clinical presentation of patients with PTC is thyroid nodule. We herein present a case of the patient initially presented with DNI, and the pathologic results finally discovered PTC.

## 2. Case Presentation

A 67-year-old male presented to the emergency department with a history of low-grade fever, difficulty swallowing, shortness of breath, and painful swelling of the left lateral neck for 1 day. He had no history of a recent upper respiratory tract infection. His medical history included well-controlled type 2 diabetes mellitus, hypertension, and dyslipidemia. On examination, his temperature was 38.8°C, heart rate was 110 beats/min, and respiratory rate was 24 times per minute. He had mild respiratory distress with inspiratory stridor. There was a tender swollen mass on the left side of the neck, measuring 5 × 6 cm.

**Figure 1:**
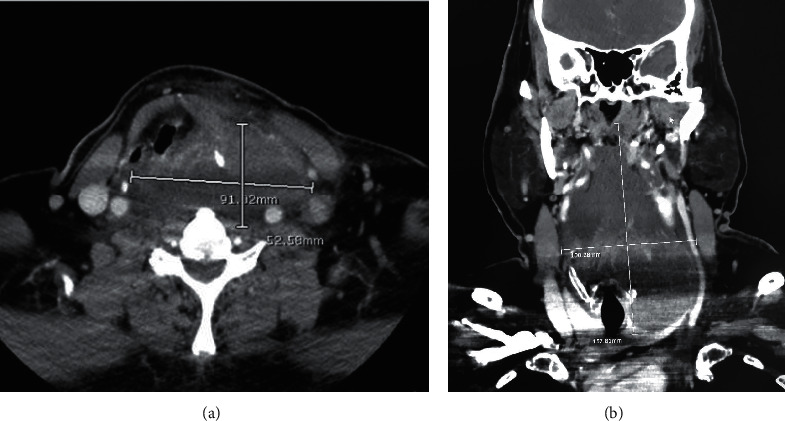
Computed tomography scan of the neck. (a) Axial and (b) coronal views showing a large lobulated partial rim-enhancing hypodense lesion with internal septation in the deep space of the left neck.

**Figure 2:**
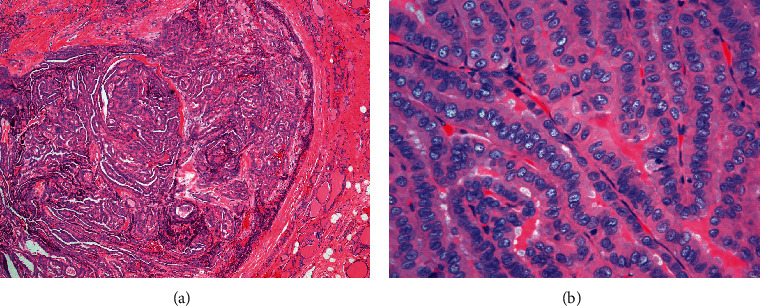
Pathology of the resected soft tissue of the neck showing (a) the presence of papillary thyroid carcinoma in the background of necrotic tissue (H&E, 20x). Pathology of the cervical lymph node showing (b) the presence of metastatic papillary thyroid carcinoma tumor (H&E, 400x).

The laboratory investigations revealed no leukocytosis (white blood count 9,000 cells/mcL). His thyroid function test showed normal free thyroxine and TSH levels. Computed tomography of the neck with contrast (Figure 1) revealed a large lobulated partially rim-enhancing hypodense lesion size (5.3 × 10 × 15.8 cm) with internal septation involving the retropharyngeal space, orohypopharynx, larynx, and left lobe of the thyroid gland. Findings were compatible with an abscess that caused adjacent airway narrowing and right lateral displacement of the trachea. Lymphadenopathy with cystic necrosis was also detected at bilateral cervical level II and left level III, measuring up to 2.8 cm in the greatest diameter. We cannot demonstrate odontogenic infection and sialadenitis. His provisional diagnosis was DNI, empirically treated with intravenous antibiotics of ceftriaxone and clindamycin. He underwent incision and drainage of the abscess. Only 10 mL of pus was drained from the left paratracheal area because the main component of the mass was the necrotic debris. Necrotic tissue samples were sent for culture and histopathology. Later, culture grew coagulase-negative staphylococci. After a 10-day course of intravenous antibiotics, his clinical signs and symptoms had improved, and he was discharged from the hospital with oral amoxicillin-clavulanic acid. However, PTC was found in the pathology of the necrotic tissue and one left cervical lymph node (Figure 2).

**Figure 3:**
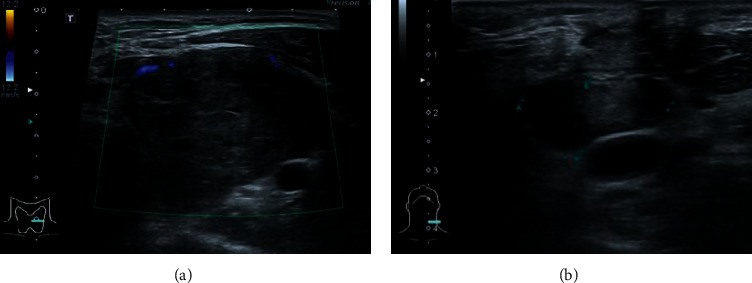
Ultrasound of the thyroid gland showing (a) a large heterogenic hypoechoic mass occupying the entire left thyroid lobe with no hypervascularity and (b) an enlarged left cervical lymph node with cystic necrosis.

Extensive preoperative imaging of the thyroidectomy was performed. After incision and drainage, ultrasound showed a 5 cm heterogenic hypoechoic mass with some cystic portion occupying the entire left thyroid lobe (Figure 3(a)). The color Doppler study showed no hypervascularity. No residual abscess was identified. Multiple enlarged left cervical lymph nodes with cystic necrosis were seen (Figure 3(b)). Barium swallowing showed a right lateral deviation of the cervical portion of the esophagus due to the compressive effect of a left neck mass. Esophagogram did not show any leak in the cervical esophagus. The fine-needle aspiration biopsy (FNAB) of the right cervical lymph node was performed, and the cytological result was reactive hyperplasia.

He underwent total thyroidectomy with the bilateral central node, left radical, and right selective neck dissection. The pathological result reported a classic PTC with tall cell features (10% of tall cells). The tumor was located on the left lobe, 3.5 × 2.3 × 1.6 cm in size with the presence of extrathyroidal and lymphovascular extension. The resected margin of the tumor was not free. Bilateral cervical lymph nodes showed metastatic PTC, predominantly in the left side. He was subsequently treated with radioactive iodine.

## 3. Discussion

Head and neck cancer and DNI are not rare. However, DNI as the initial presentation of head and neck cancer is uncommon. The prevalence ranges from 1.0% to 5.6% [2, 5–7]. Well-differentiated thyroid cancer such as PTC typically presents as a thyroid nodule. To date, only one case of DNI as the first manifestation of PTC has been reported in the literature [2].

The pathogenesis of PTC presenting as DNI is unclear. It has been postulated that the central necrosis of cystic cervical metastasis may be susceptible to infection because of tumor necrosis due to insufficient blood supply. A component of tall cell PTC which is aggressive in behavior may contribute to the large size of tumor and lymph nodes [8]. Another potential mechanism is the infected ulcer of the primary tumor which drains to the lymph node and forms an abscess [9]. The source of infection may arise from the mandibular teeth, tonsils, parotid gland, middle ear, or sinuses. In addition, immunocompromised status (e.g., diabetes mellitus and poor oral hygiene) may predispose cystic metastases to infection [10, 11].

DNI often has a rapid progression to life-threatening complications. The mainstays of treatment for DNI are airway maintenance, fluid resuscitation, effective antibiotic therapy, and surgical drainage when indicated. The percentage of cancers in patients with DNI is extremely low. However, cystic lymphadenopathy contralateral to the site of infection and a large solid necrotic component of mass should raise the suspicion of neoplasm in this case. To distinguish between the infectious process and malignancy, FNAB should be performed. However, cytological examination of the cystic lesions of malignancy might yield a false-negative result because of the dilutional effect of cystic fluid. Routine pathological examination of the abscess tissue is recommended in all cases with DNI, especially in those over 40 years of age [2, 7, 9, 12]. If the neck swelling diminishes, repeated FNAB, endoscopic examination of the upper aerodigestive tract, or imaging study should be considered.

In conclusion, although well-differentiated thyroid cancer presenting as DNI is very rare, physicians should recognize the possibility of PTC. Careful evaluation of head and neck cancer including thyroid cancer may be required.

## Data Availability

The data used to support the findings of this study are available from the corresponding author upon request.

## References

[1] Gujrathi A. B., Ambulgekar V., Kathait P. (2016). Deep neck space infection—a retrospective study of 270 cases at tertiary care center. *World Journal of Otorhinolaryngology Head and Neck Surgery*.

[2] Lin Y. Y., Hsu C. H., Lee J. C. (2012). Head and neck cancers manifested as deep neck infection. *European Archives of Oto-Rhino-Laryngology*.

[3] Soon S. R., Kanagalingam J., Johari S., Yuen H. W. (2012). Head and neck cancers masquerading as deep neck abscesses. *Singapore Medical Journal*.

[4] Ito Y., Miyauchi A., Kihara M., Fukushima M., Higashiyama T., Miya A. (2018). Overall survival of papillary thyroid carcinoma patients: a single-institution long-term follow-up of 5897 patients. *World Journal of Surgery*.

[5] Ridder G. J., Technau-Ihling K., Sander A., Boedeker C. C. (2005). Spectrum and management of deep neck space infections: an 8 year experience of 234 cases. *Otolaryngology-Head and Neck Surgery*.

[6] Wang L. F., Kuo W. R., Tsai S. M., Huang K. J. (2003). Characterizations of life-threatening deep cervical space infections: a review of one hundred ninety-six cases. *American Journal of Otolaryngology*.

[7] Wang C. P., Ko J. Y., Lou P. J. (2006). Deep neck infection as the main initial presentation of primary head and neck cancer. *The Journal of Laryngology & Otology*.

[8] Oh W. J., Lee Y. S., Cho U. (2014). Classic papillary thyroid carcinoma with tall cell features and tall cell variant have similar clinicopathologic features. *Korean Journal of Pathology*.

[9] Chen W. T., Lee J. W., Hsieh K. W., Chen R. F. (2014). Deep neck abscess as the predominant initial presentation of carcinoma of unknown primary: a case report. *Oncology Letters*.

[10] Chen M. K., Wen Y. S., Chang C. C., Lee H. S., Huang M. T., Hsiao H. C. (2000). Deep neck infections in diabetic patients. *American Journal of Otolaryngology*.

[11] Huang T. T., Tseng F. Y., Liu T. C., Hsu C. J., Chen Y. S. (2005). Deep neck infection in diabetic patients: comparison of clinical picture and outcomes with nondiabetic patients. *Otolaryngology-Head and Neck Surgery*.

[12] Karaman E., Duman C., Mercan H., Ozaras R., Cansız H. (2009). Relapsing deep neck infection may indicate a coexisting esophagus cancer. *Kulak Burun Bogaz Ihtisas Dergisi: KBB =  Journal of Ear, Nose, and Throat*.

